# Comparison of PANI- and PEDOT:PSS-Incorporated GelMA Hydrogels for Neuronal Differentiation of SH-SY5Y Cells

**DOI:** 10.3390/gels12100892

**Published:** 2026-10-02

**Authors:** Gayeon Pyo, Ha Som Yu, Yeontae Kang, Hansol Lee, Hyun Jong Lee

**Affiliations:** School of Chemical, Biological and Battery Engineering, Gachon University, 1342 Seongnam-daero, Seongnam-si 13120, Gyeonggi-do, Republic of Korea

**Keywords:** conductive hydrogel, PEDOT:PSS, neural tissue engineering, neurite outgrowth, photocrosslinking

## Abstract

Conductive hydrogels are widely pursued for neural tissue engineering, but the conventional route of polymerizing conductive monomers in situ requires toxic monomers or organic solvents and is incompatible with cell encapsulation. Here, conductive hydrogels were prepared in a single step by dispersing pre-polymerized polyaniline (PANI) particles or poly(3,4-ethylenedioxythiophene):poly(styrene sulfonate) (PEDOT:PSS) nanoparticles into a gelatin methacryloyl (GelMA) precursor and photocrosslinking, giving a simpler, cell-friendly route from an injectable precursor; the two polymers were compared directly within a single platform. PEDOT:PSS was uniformly integrated, whereas PANI progressively aggregated, and these differences in dispersibility were reflected in the microstructure, mechanical properties, and swelling behavior of the hydrogels. Both polymers increased the electrical conductance of the hydrogels; the increase was statistically significant for PANI but small in magnitude, and the cellular outcomes diverged in the opposite direction. Over 7 days, the PEDOT:PSS 0.2% group supported SH-SY5Y metabolic activity comparable to GelMA, whereas PANI reduced it. After neuronal induction, neurite outgrowth appeared most extensive in the PEDOT:PSS 0.2% hydrogels, with co-detection of βIII-tubulin, MAP2, and synaptophysin. Cells could also be encapsulated within the precursor and photocrosslinked in situ, remaining viable in all groups, demonstrating that the one-step route is compatible with three-dimensional cell-laden constructs. Because the conductance differences were small and ran opposite to the biological outcome, these findings indicate that the performance of GelMA-based conductive hydrogels is associated with polymer dispersibility, surface chemistry, and mechanical properties rather than with bulk conductance, and point to dispersion stability rather than intrinsic conductivity as the primary design variable at cell-compatible loadings, with GelMA/PEDOT:PSS as a candidate platform for neural tissue engineering.

## 1. Introduction

Neurodegenerative diseases and acute neurological injuries cause chronic pain along with impairments in motor and sensory functions, often resulting in long-term functional loss that substantially reduces quality of life. Conventional pharmacological treatments are limited in their efficacy, as many therapeutic agents have difficulty crossing the blood–brain barrier, and even when they reach the target site, their effects are generally restricted to symptom relief rather than complete restoration of damaged neural function. Surgical approaches such as autologous or allogeneic nerve grafting have also been explored; however, their practical clinical application remains challenging due to additional tissue damage at the harvest site, limited tissue availability, and the risk of immune rejection [1]. Against this background, hydrogel-based scaffolds have attracted increasing attention in neural tissue engineering as platforms capable of providing a three-dimensional microenvironment that mimics the extracellular matrix, thereby supporting neuronal growth, survival, and differentiation [2,3]. Among the various hydrogel materials investigated, gelatin methacryloyl (GelMA), synthesized by introducing methacrylate groups into gelatin, a collagen-derived denatured protein, has emerged as a particularly promising candidate. GelMA retains the inherent biological properties of gelatin, including RGD sequences that promote cell adhesion and migration and MMP-sensitive motifs that enable cell-mediated matrix remodeling. Furthermore, its mechanical properties can be readily tuned to match those of various soft tissues, including neural tissue, by adjusting the GelMA concentration and photocrosslinking conditions [4,5]. Despite these advantages, GelMA is intrinsically electrically insulating, which limits its ability to recapitulate the electroactive nature of neural tissue, an important factor associated with neuronal signaling, differentiation, and functional maturation [6,7].

To address this limitation, conductive hydrogels have been developed by incorporating electrically active materials such as polyaniline (PANI), polypyrrole (PPy), and poly(3,4-ethylenedioxythiophene):poly(styrene sulfonate) (PEDOT:PSS) into hydrogel matrices, providing both ionic and electronic conductivity and a more biomimetic interface for electroactive tissues [8,9]. In conventional approaches, however, a hydrogel scaffold is first formed and conductive monomers are subsequently introduced into its interior or surface and polymerized in situ [10,11]. For example, Wu et al. generated a GelMA-PANI composite by infiltrating preformed GelMA disks with aniline and oxidant solutions and polymerizing PANI within the matrix through interfacial in situ polymerization [10]. Although such strategies can achieve high electrical conductivity, the use of toxic monomers, organic solvents, and harsh oxidants, together with the extensive washing required to remove residual reactants, is inherently incompatible with the presence of living cells. Consequently, in situ polymerization is restricted to acellular scaffolds onto which cells can only be seeded after fabrication, and cannot be used to encapsulate cells within the conductive matrix during gelation, which is a key requirement for building three-dimensional neural constructs.

An alternative that circumvents this constraint is to disperse pre-polymerized conductive polymer particles directly into the hydrogel precursor solution and complete gelation in a single photocrosslinking step. Because no monomer, oxidant, or organic solvent is involved during gelation, this one-step strategy is intrinsically cell-friendly and, in principle, compatible with in situ crosslinking in the presence of cells, offering a route toward cell-laden conductive constructs that conventional in situ polymerization cannot support. Within this framework, the choice of conductive polymer becomes more than a question of conductivity alone, because each polymer introduces distinct physicochemical characteristics, including dispersibility, surface chemistry, and the resulting mechanical behavior, that may shape the biological microenvironment as strongly as conductivity itself. PANI and PEDOT:PSS are particularly relevant candidates for this particle-dispersion approach, as both can be prepared in dispersible particulate form yet differ markedly in their colloidal and surface properties. PANI offers a straightforward synthesis, good environmental stability, and redox- and pH-tunable conductivity [12], whereas PEDOT:PSS is commercially available as a stable aqueous dispersion well suited to water-based hydrogel processing and exhibits relatively favorable biocompatibility [13].

Despite the appeal of this approach, prior work has examined these polymers only in isolation and within different platforms. Spencer et al. dispersed PEDOT:PSS into a GelMA precursor and photocrosslinked the composite, demonstrating that it supported the survival and spreading of encapsulated myoblasts [14], and Zhang et al. reported that a related GelMA/PEDOT:PSS system supported neural stem cell viability and neuronal differentiation [15]. To our knowledge, however, pre-polymerized PANI has not been dispersed into GelMA in particulate form without in situ polymerization, and PANI and PEDOT:PSS have not been compared directly within a single one-step GelMA platform. Moreover, existing studies have largely assessed cell survival and spreading rather than the progression of neuronal differentiation, leaving open the question of how the choice of conductive polymer, and the material properties it imparts, influences neuronal commitment and maturation.

Therefore, this study examines how the type of conductive polymer dispersed into a one-step photocrosslinkable GelMA hydrogel shapes the biological microenvironment and the neuronal differentiation of SH-SY5Y cells. Pre-polymerized PANI particles or PEDOT:PSS nanoparticles were dispersed into a GelMA precursor solution and gelated in a single UV photocrosslinking step, avoiding the toxic monomers, organic solvents, and extensive washing required by in situ polymerization, and the injectable precursor solution further points toward future cell-laden, in situ crosslinkable applications. Within this platform, PANI and PEDOT:PSS were compared in terms of their dispersion characteristics, microstructural, mechanical, and swelling behaviors, and their effects on the viability and neuronal differentiation of SH-SY5Y cells. By relating these material properties to the resulting cellular responses, we aimed to identify which characteristics of the incorporated polymer are most strongly associated with neuronal differentiation, and to assess whether such one-step fabricated conductive hydrogels can serve as a practical and cell-compatible platform with potential for neural tissue engineering applications.

## 2. Results and Discussion

### 2.1. Fabrication and Characterization of Conductive Hydrogels

**Figure 1:**
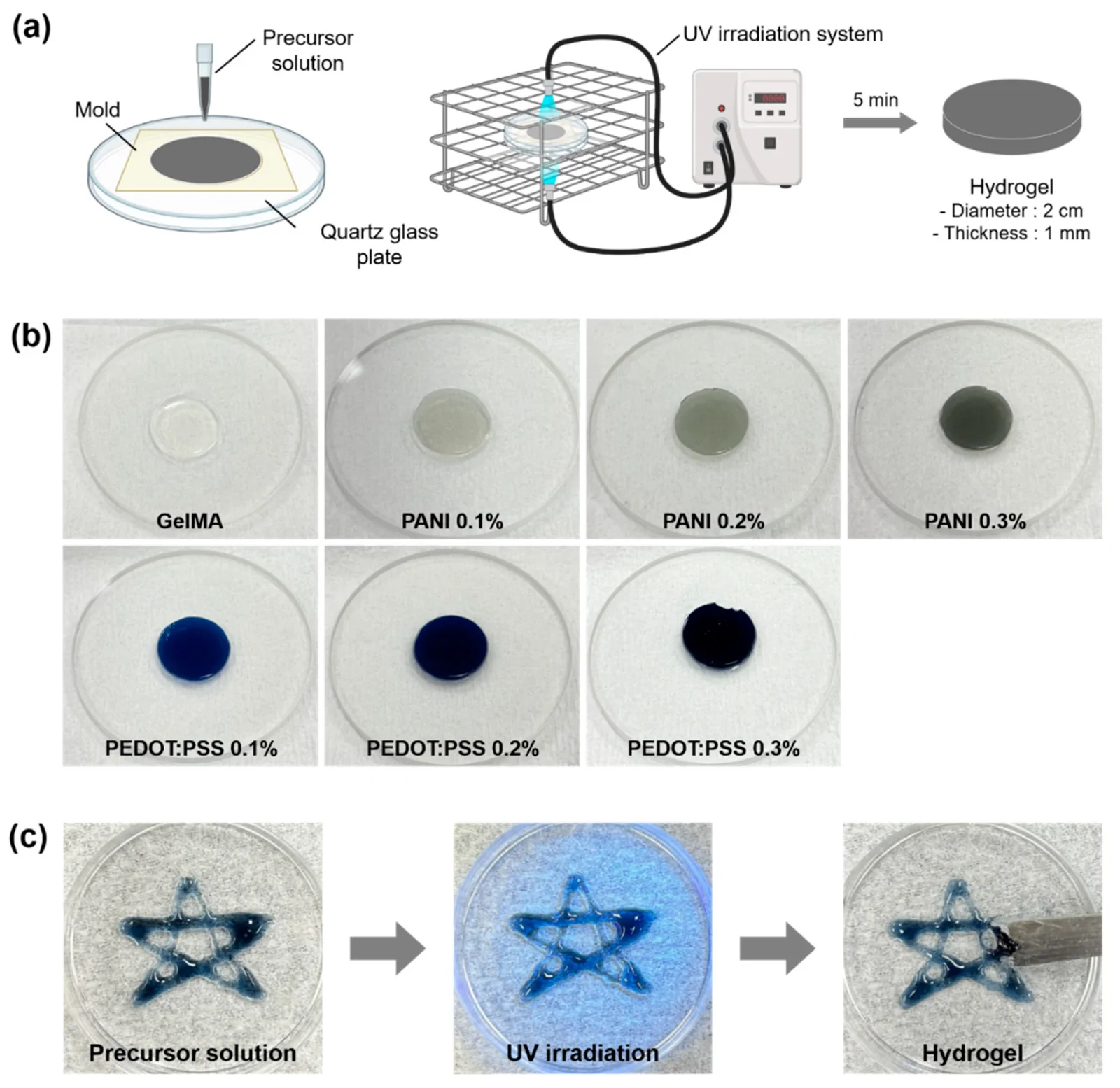
Fabrication of conductive GelMA hydrogels. (**a**) Schematic illustration of disk-shaped conductive hydrogel fabrication via one-step UV photocrosslinking. (**b**) Gross appearance of conductive hydrogels. (**c**) Injectable property of the conductive hydrogel precursor solution demonstrated by extrusion through a syringe followed by UV photocrosslinking.

Conductive hydrogels were fabricated through a one-step process in which pre-polymerized PANI particles or PEDOT:PSS nanoparticles were directly dispersed into a GelMA precursor solution, followed by UV photocrosslinking. A schematic overview of the fabrication process is presented in Figure 1a. Macroscopic observation revealed that the color of the fabricated hydrogels became progressively darker with increasing conductive polymer concentration in both groups (Figure 1b). Notably, whereas PEDOT:PSS appeared homogeneously integrated within the hydrogel matrix, visible particle aggregation was apparent in the PANI groups upon naked-eye inspection, indicating that the two polymers differ markedly in their dispersibility within GelMA. As shown in Figure 1c, the precursor solution could also be readily extruded through a syringe and photocrosslinked into a freestanding structure, illustrating that gelation occurs in a single step from an injectable precursor.

**Figure 2:**
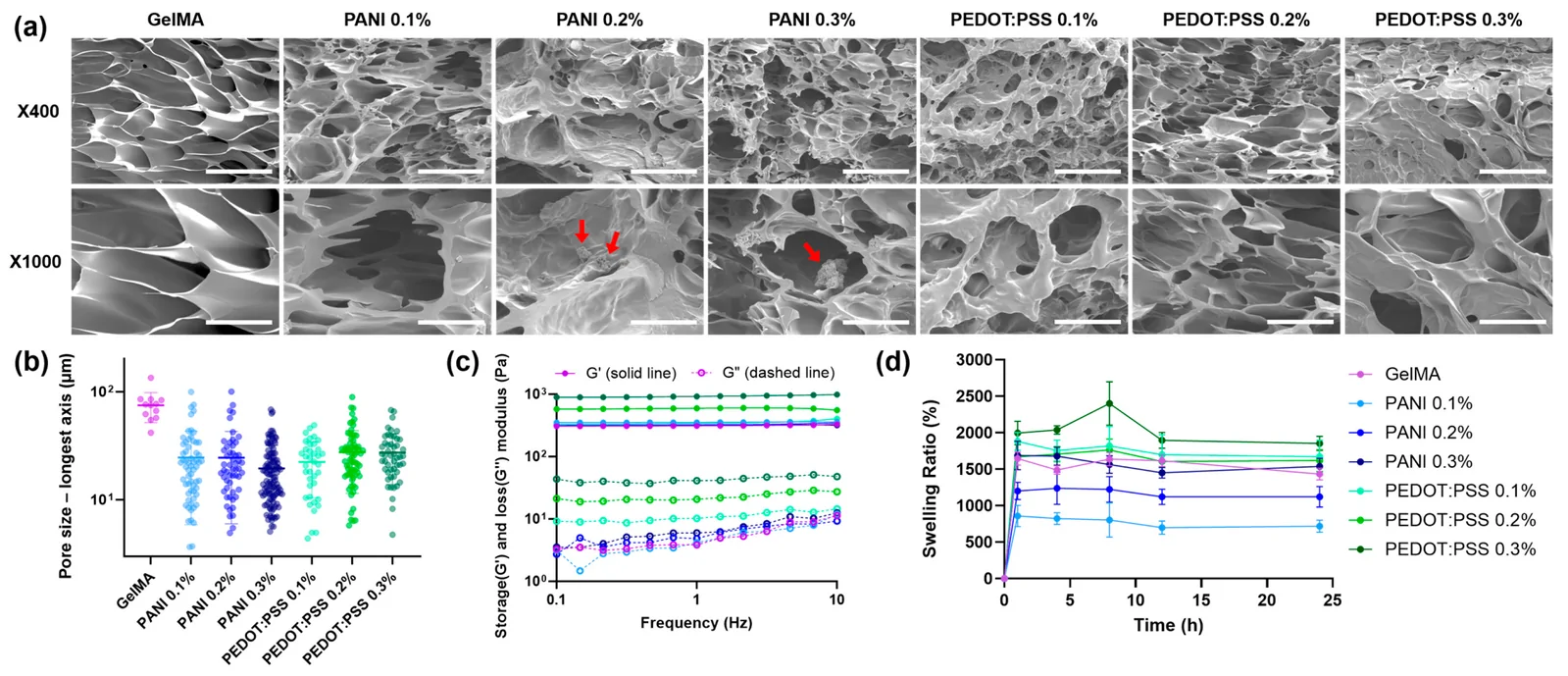
Structural and mechanical characterization of conductive GelMA hydrogels. (**a**) SEM images of freeze-dried hydrogel cross-sections (scale bar: 100 µm at × 400; 50 µm at × 1000); red arrows indicate features consistent with particle aggregates. (**b**) Pore size of the freeze-dried hydrogels measured from the ×400 SEM images (dots, individual pores; horizontal bars, mean ± SD). The numbers of pores analyzed were 12, 62, 54, 116, 38, 71 and 50 for GelMA; PANI 0.1%, 0.2% and 0.3%; and PEDOT:PSS 0.1%, 0.2% and 0.3%, respectively. (**c**) Storage modulus (G′) and loss modulus (G″) of hydrogels measured by frequency sweep rheometry at 37 °C. (**d**) Swelling ratio of hydrogels immersed in DPBS at 37 °C over time.

These differences in dispersibility were reflected in the internal microstructure of the hydrogels. SEM imaging of freeze-dried cross-sections revealed distinct structural differences among the groups (Figure 2a). The GelMA group exhibited a uniform porous structure with well-interconnected networks. In the PANI groups, the porous morphology became increasingly heterogeneous with rising concentration, and features consistent with particle aggregates were identified at concentrations of 0.2% and above, as indicated by the red arrows. In contrast, the PEDOT:PSS groups retained well-developed and comparatively regular porous structures across the concentration range examined. These observations were confirmed by quantitative analysis of the pore size (Figure 2b). The GelMA group had markedly larger pores than every polymer-containing group, whereas the polymer-containing groups differed little from one another. The pore-size distributions were nevertheless consistently narrower in the PEDOT:PSS groups than in the PANI groups, consistent with the more uniform particle dispersion.

Rheological analysis showed that all hydrogel groups exhibited storage modulus (G′) values exceeding loss modulus (G″) across the measured frequency range, confirming stable gel formation (Figure 2c); the mean moduli and the corresponding loss tangents are summarized in Table 1. In the PANI groups, G′ remained largely unchanged with increasing concentration, staying close to the GelMA value, and the loss tangent was similarly unaffected. In contrast, the PEDOT:PSS groups showed a concentration-dependent increase in both G′ and the loss tangent, the PEDOT:PSS 0.2% group being approximately 1.9-fold stiffer than the GelMA group and the corresponding PANI 0.2% group. Swelling analysis showed that all groups exhibited the greatest increase in swelling ratio during the initial 1 h, after which equilibrium was reached (Figure 2d). In the PANI groups, the equilibrium swelling ratio was lowest at 0.1% and increased progressively with concentration, whereas the PEDOT:PSS groups showed a marked concentration-dependent increase.

**Figure 3:**
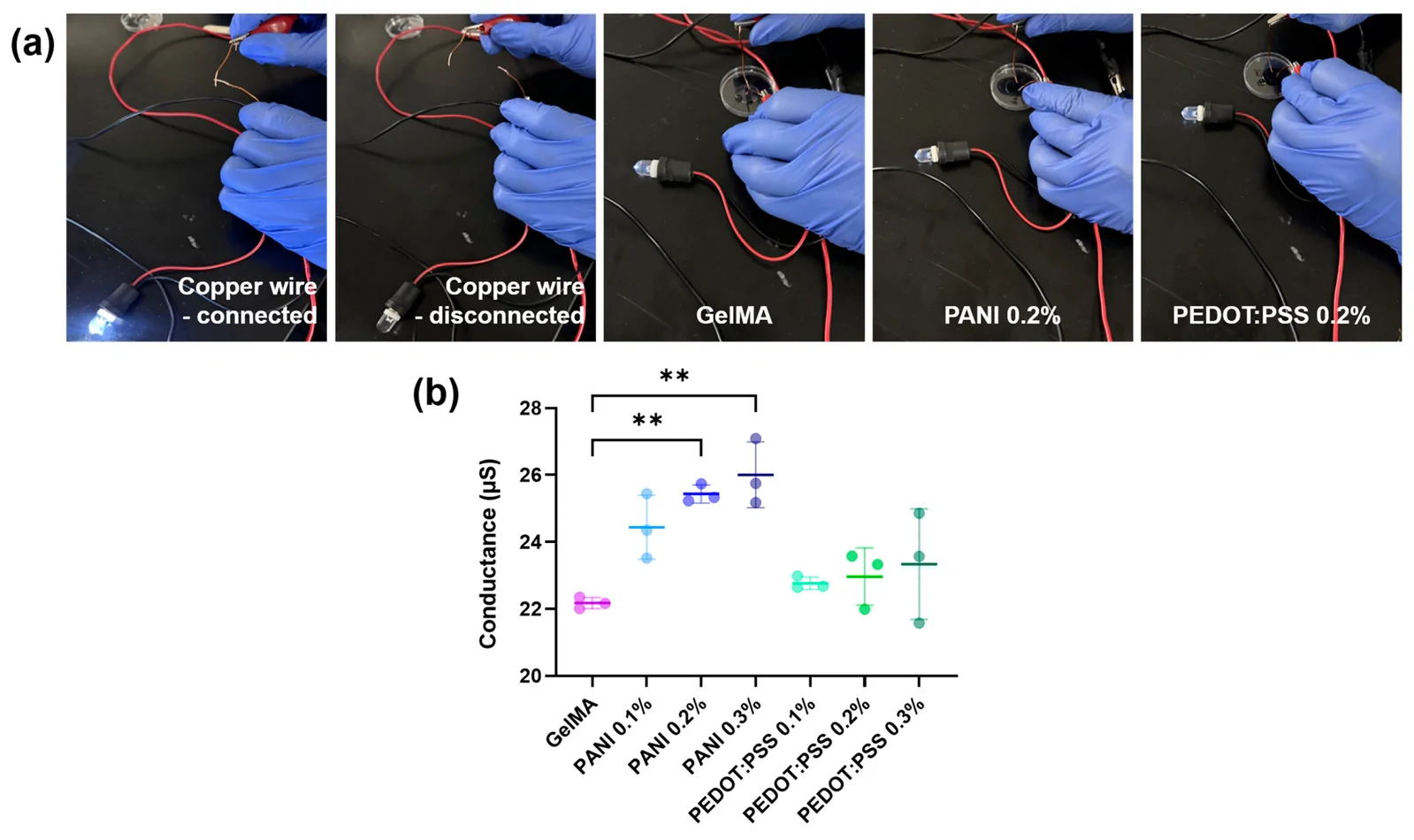
Electrical characterization of conductive GelMA hydrogels. (**a**) Photographs of LED illumination tests for qualitative assessment of electrical conductivity. (**b**) Electrical conductance of the hydrogels (colors distinguish the groups labeled on the x-axis; dots, individual specimens; horizontal bars, mean ± SD; *n* = 3 per group; ** *p* < 0.01 versus GelMA).

Qualitative assessment using an LED illumination circuit confirmed that all hydrogel groups were capable of conducting electricity (Figure 3a). The electrical properties were then compared quantitatively by measuring the conductance of the hydrogels (Figure 3b and Table 2). Both polymers raised the conductance of the hydrogels relative to GelMA, but the effect depended on which polymer was incorporated. The increase was significant for PANI at 0.2% and 0.3%, whereas none of the PEDOT:PSS groups differed significantly from GelMA. **No difference between concentrations within either polymer reached significance.** The PANI groups were accordingly the more conductive of the two, with PANI 0.3% differing significantly from all three PEDOT:PSS groups. The magnitude of the change was nevertheless modest, the conductance of every group remaining of the same order as that of GelMA. Thus, although the incorporated polymers produced a measurable increase in conductance, the groups diverged far more substantially in dispersibility, microstructure, mechanical stiffness, and swelling behavior. We therefore proceeded to examine how these material differences would influence the viability and neuronal differentiation of SH-SY5Y cells.

### 2.2. Cytocompatibility of Conductive Hydrogels

**Figure 4:**
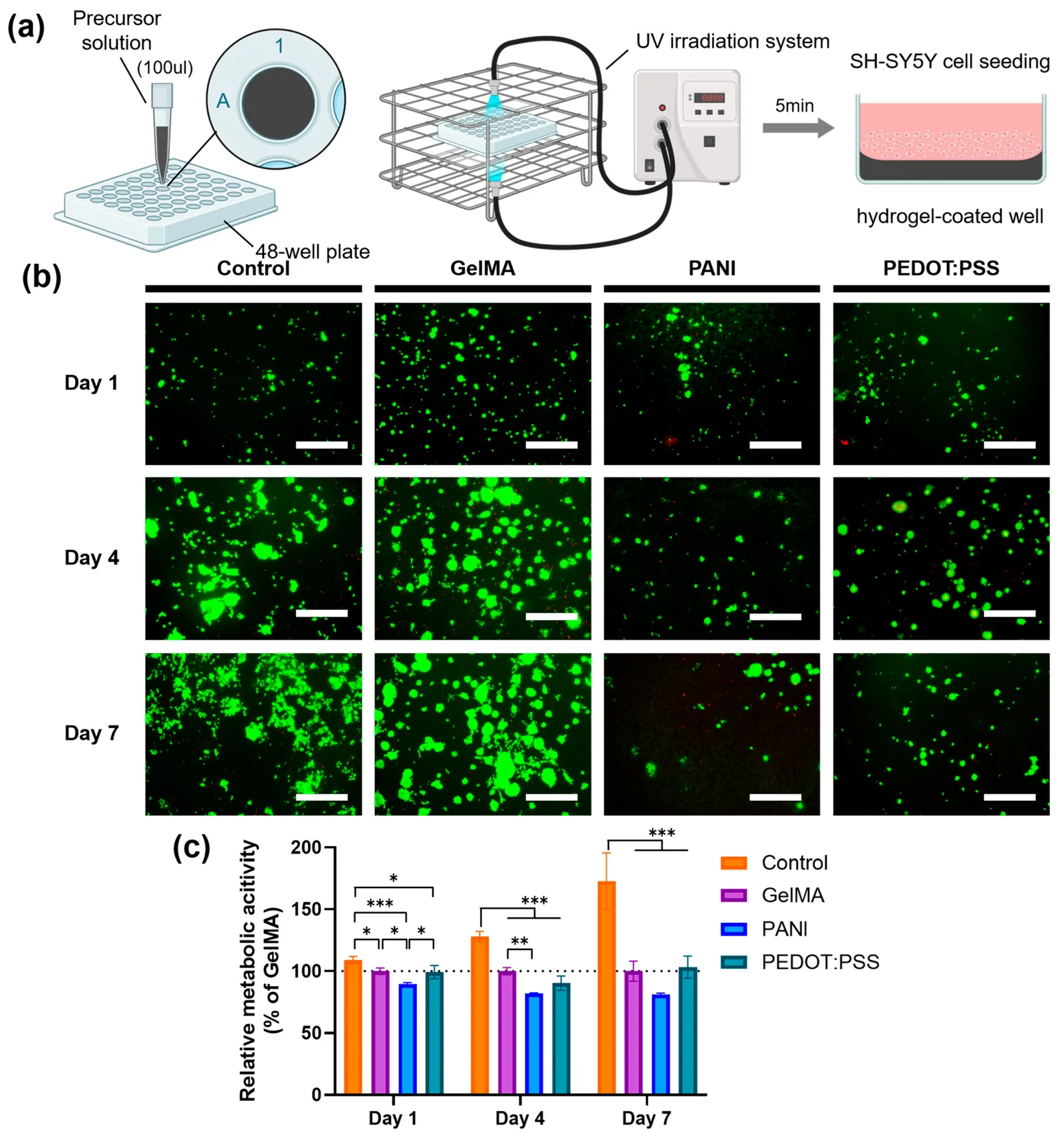
In vitro cytocompatibility of conductive GelMA hydrogels with SH-SY5Y cells. (**a**) Schematic illustration of coating-type conductive hydrogel preparation for 2D cell culture. (**b**) Live/Dead staining images of SH-SY5Y cells cultured on conductive hydrogels on days 1, 4, and 7 (scale bar: 750 µm; green: live cells; red: dead cells). (**c**) Metabolic activity of SH-SY5Y cells assessed by CCK-8 assay on days 1, 4, and 7, expressed as a percentage of the GelMA group (* *p* < 0.05, ** *p* < 0.01, *** *p* < 0.001).

A key practical advantage of the one-step photocrosslinking strategy employed in this study is the elimination of toxic monomers and organic solvents from the fabrication process. Based on the physicochemical characterization, the 0.2% concentration was selected for all subsequent cell studies; it provided well-developed, uniform hydrogel structures, whereas higher concentrations increased the opacity of the hydrogels to a degree that could interfere with downstream cell imaging. To verify that this approach yields a genuinely cell-compatible culture environment, the cytocompatibility of the conductive hydrogels toward SH-SY5Y cells was assessed using Live/Dead staining and CCK-8 assays over a 7-day culture period. Hydrogels were prepared in a coating format on 48-well plates, and a schematic of the experimental setup is shown in Figure 4a.

Live/Dead staining images acquired on days 1, 4, and 7 are shown in Figure 4b. In the tissue culture plate (TCP) control and GelMA groups, a generally high proportion of viable cells was maintained throughout the culture period, with cells in the GelMA group tending to form clustered morphologies. In the PANI 0.2% group, the density of viable cells was visibly lower than in the other groups throughout the culture period. A small number of cells showed a weak red signal on day 1, likely reflecting stressed but still living cells rather than overt cell death, and few clearly dead cells were observed at later time points, as nonviable cells generally detached from the surface and were removed during washing. The PEDOT:PSS 0.2% group contained noticeably fewer viable cells than the GelMA group, but clearly more than the PANI 0.2% group. Overall, these observations indicate that PANI incorporation suppressed cell growth on the hydrogel surface rather than causing pronounced cytotoxicity.

Metabolic activity measured by the CCK-8 assay is presented in Figure 4c as a percentage of the GelMA group at the same time point. Metabolic activity increased gradually over the culture period in all groups. The TCP control group exhibited the highest activity throughout. Among the hydrogel groups, the PANI 0.2% group showed the lowest metabolic activity. On days 1 and 4, this activity was significantly lower than that of the GelMA group, whereas the GelMA and PEDOT:PSS 0.2% groups did not differ significantly at any time point. By day 7, the differences among the hydrogel groups were no longer statistically significant, and all hydrogel groups remained significantly lower than the TCP control throughout the culture period (Figure 4c). Because each Live/Dead image samples a single field, in which the clustered growth of cells on GelMA produces locally dense regions, whereas the CCK-8 assay integrates the whole well, the cell density seen in a given field is not expected to track the whole-well metabolic activity closely. Taken together with the Live/Dead images, in which few clearly dead cells were observed in any hydrogel group, these results indicate that PANI incorporation limited the accumulation of metabolically active cells rather than killing the cells already present. Thus, the two polymers exerted distinctly different effects on SH-SY5Y behavior, with PANI consistently yielding the lowest metabolic activity and PEDOT:PSS supporting activity comparable to GelMA.

### 2.3. Neuronal Differentiation of SH-SY5Y Cells on Conductive Hydrogels

**Figure 5:**
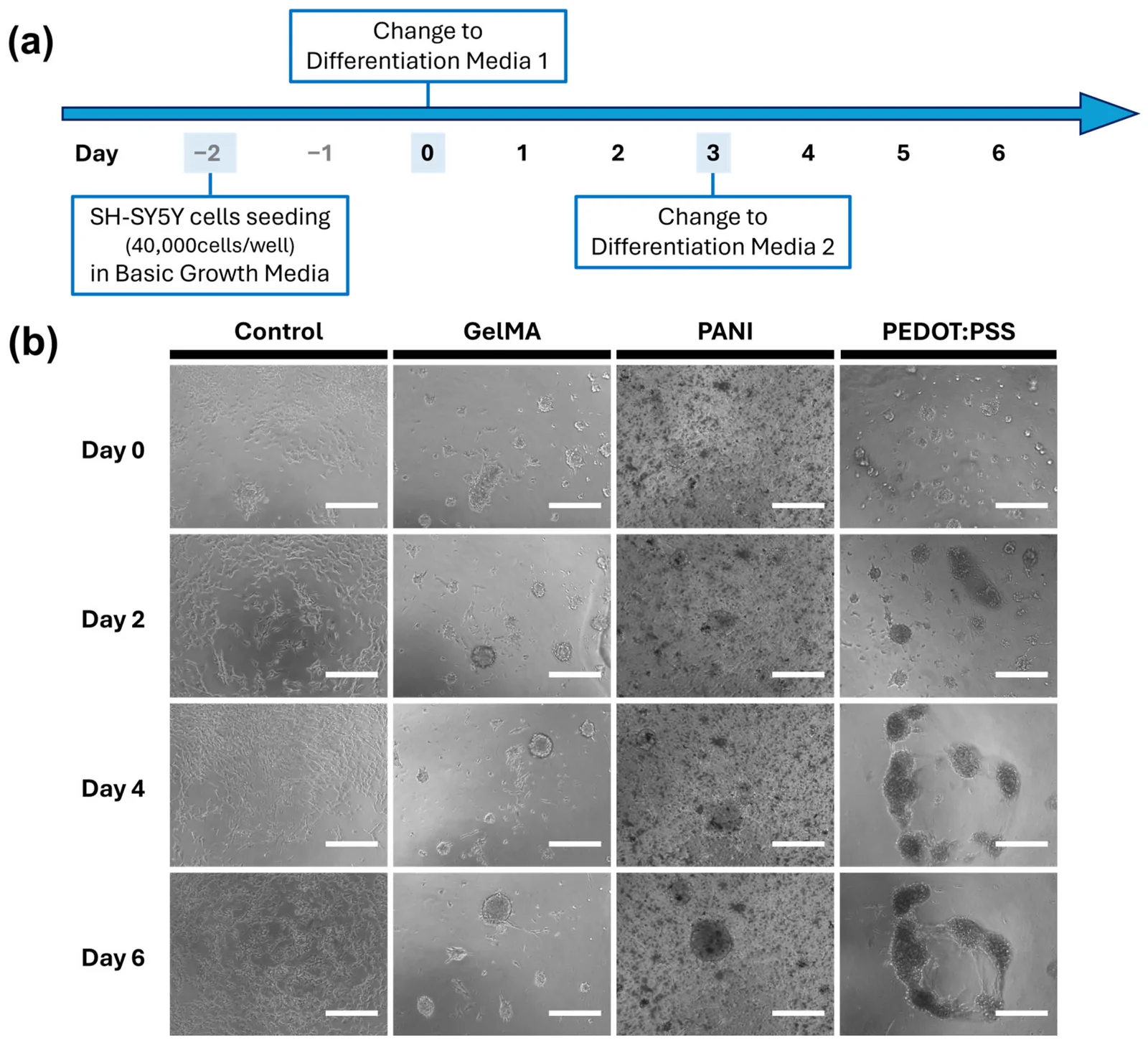

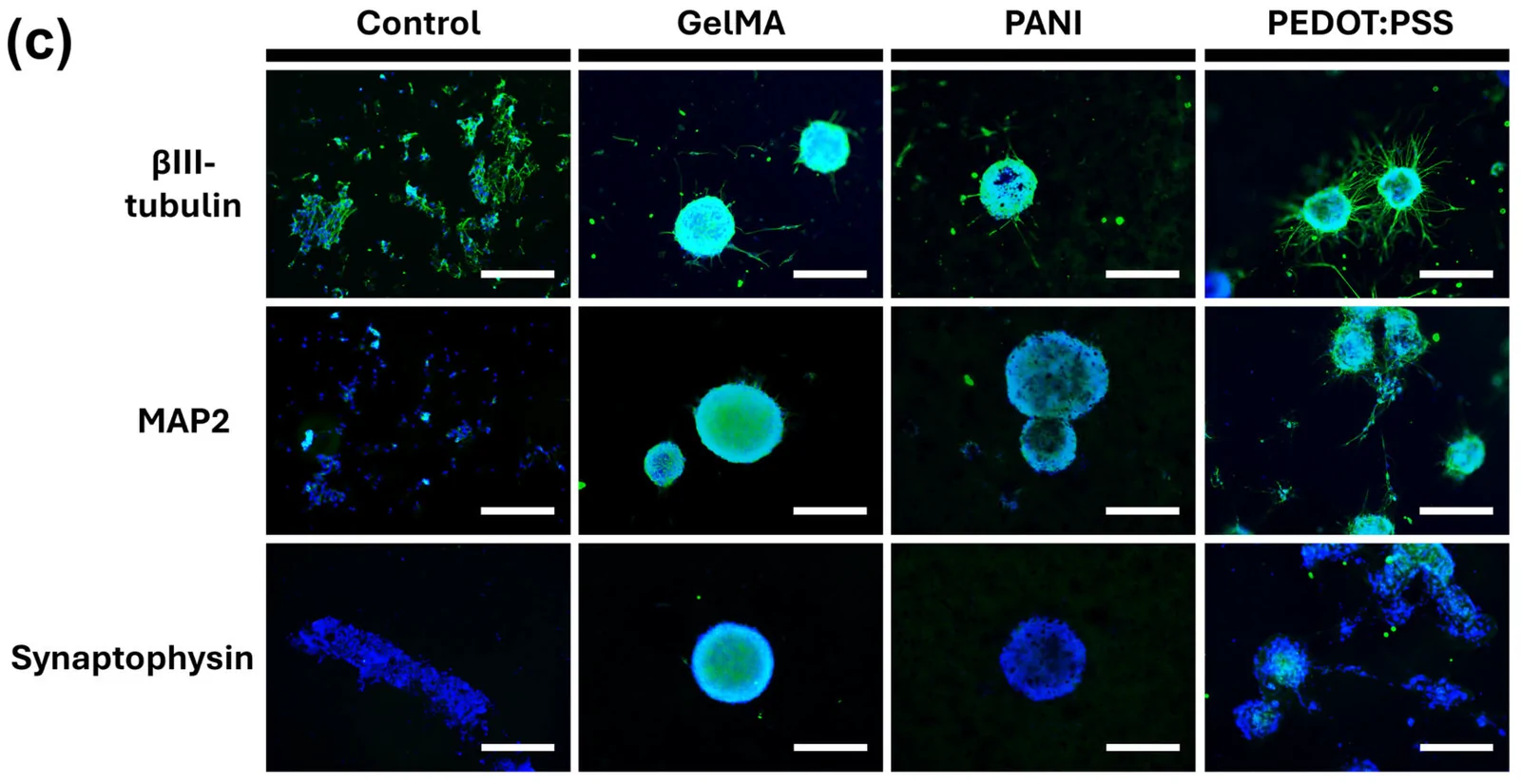
Neuronal differentiation of SH-SY5Y cells on conductive GelMA hydrogels. (**a**) Schematic timeline of the differentiation protocol. (**b**) Bright-field microscopy images of SH-SY5Y cells during differentiation on days 0, 2, 4, and 6 (scale bar: 300 µm). (**c**) Immunofluorescence staining images of βIII-tubulin, MAP2, and synaptophysin in differentiated SH-SY5Y cells on day 6 (scale bar: 300 µm).

Differentiation was induced using a sequential two-stage protocol (Figure 5a). Morphological changes during the 6-day differentiation period are shown in Figure 5b. In the TCP control group, thin neurite-like extensions connecting adjacent cells were observed alongside an increase in cell density over time. In all hydrogel groups, cells adopted clustered morphologies. In the PEDOT:PSS 0.2% group, distinct neurite-like extensions were observed surrounding cell clusters from early in the differentiation period, and by day 4, neurites from different cell aggregates began interconnecting to form network-like structures. Neurite-like extensions were also observed in the GelMA and PANI 0.2% groups, but tended to be shorter and less interconnected than those in the PEDOT:PSS 0.2% group.

Neuronal marker expression was examined by immunofluorescence staining on day 6 of differentiation (Figure 5c). Three markers representing distinct aspects of the neuronal phenotype were evaluated at this single time point: βIII-tubulin, an early neuronal cytoskeletal marker; MAP2, associated with dendritic maturation; and synaptophysin, a presynaptic vesicle protein. In the TCP control group, positive βIII-tubulin signals were observed; however, dense neurite overlap precluded clear assessment of individual directionality and connectivity. Because the polymer-containing hydrogels were dark and opaque, marker expression was interpreted from signal localization and neurite morphology rather than from absolute fluorescence intensity.

In all hydrogel groups, the βIII-tubulin signal was observed in the cell aggregates. In the GelMA and PANI 0.2% groups, only short processes extended from the aggregates, whereas in the PEDOT:PSS 0.2% group, βIII-tubulin was distributed throughout markedly elongated neurites that interconnected adjacent aggregates. MAP2 showed the same pattern: signal was present in the aggregates of all groups, but elongated MAP2-positive neurite-like structures clearly distinguished the PEDOT:PSS 0.2% group from the GelMA and PANI groups, in which the processes remained very short. Synaptophysin, which generally gave a weaker signal than the other two markers, was localized mainly to the aggregates in the GelMA and PANI 0.2% groups, whereas in the PEDOT:PSS 0.2% group it was additionally observed at regions of cell–cell contact and along neurite projections.

For reference, the neurites in the representative βIII-tubulin image of each group were also counted and measured (Figure S2); because these values derive from a single field per group, they are provided only as a description of the representative images and were not used for comparison between groups. Overall, neurite outgrowth appeared most extensive in the uniformly dispersed PEDOT:PSS 0.2% hydrogels, even though the PANI 0.2% group was the more conductive of the two.

### 2.4. Three-Dimensional Encapsulation of SH-SY5Y Cells

**Figure 6:**
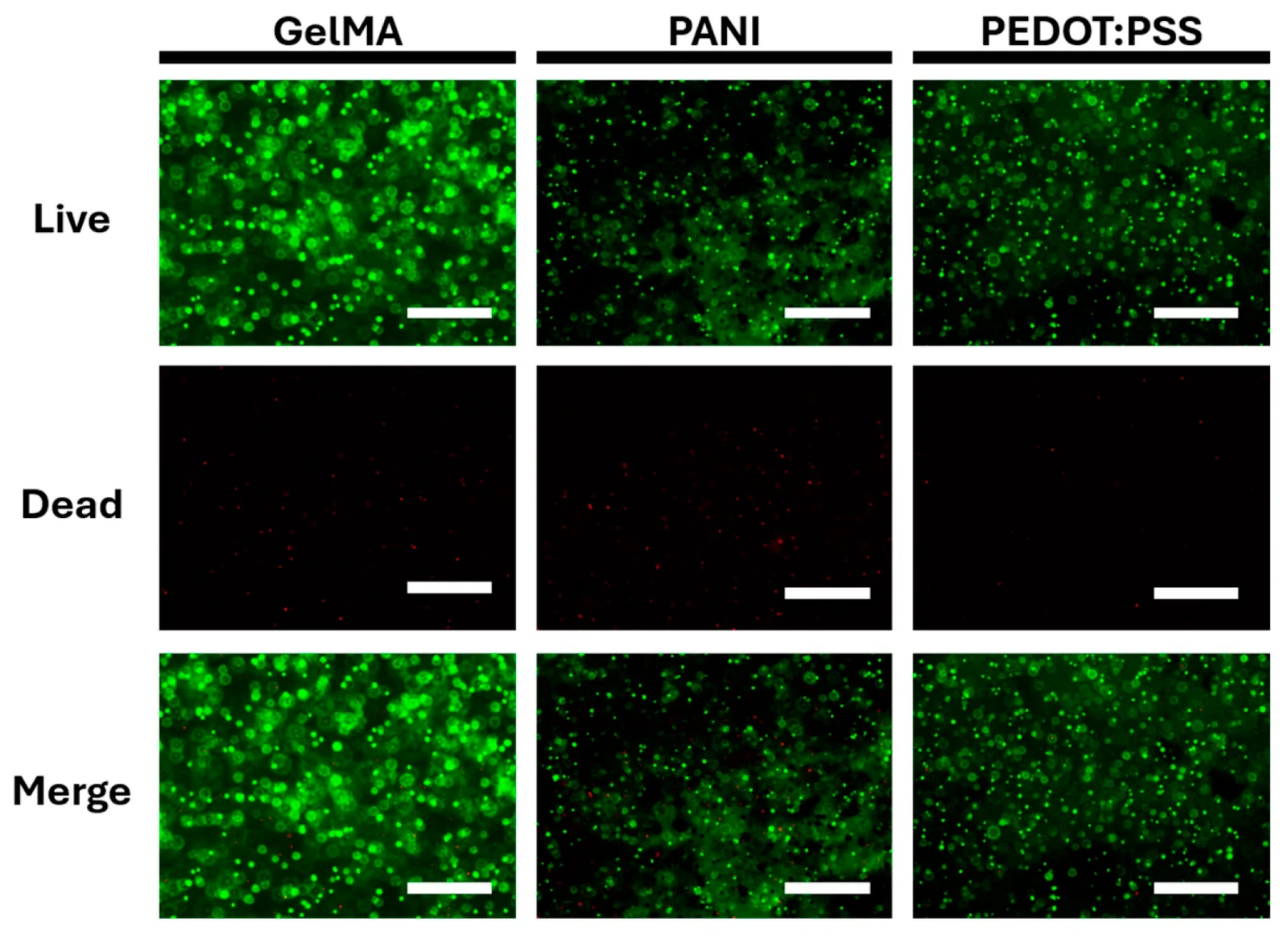
Three-dimensional encapsulation of SH-SY5Y cells in conductive GelMA hydrogels. Live/Dead staining images of SH-SY5Y cells encapsulated within the hydrogels and photocrosslinked in situ, imaged after 24 h of culture (scale bar: 300 µm; green: live cells; red: dead cells).

A practical motivation for dispersing pre-polymerized particles rather than polymerizing conductive monomers in situ is that gelation can be carried out in the presence of cells. To test this directly, SH-SY5Y cells were suspended in the precursor solution and photocrosslinked in situ, and the resulting cell-laden constructs were stained after 24 h (Figure 6). Viable cells were distributed throughout the constructs in all three groups and dead cells were sparse. These results show that the one-step dispersion route is compatible with cell encapsulation and in situ photogelation, and that the conductive hydrogels can therefore be used as cell-laden three-dimensional constructs rather than only as preformed substrates for surface seeding.

### 2.5. Discussion

The structural differences between PANI and PEDOT:PSS observed in this study are attributable to their distinct physicochemical characteristics. PANI exhibits a strong intrinsic tendency toward aggregation and crystallization due to π-π stacking interactions and interchain forces along its conjugated backbone [16,17]. Although CSA doping was employed to improve dispersibility, the long-term stability of PANI in aqueous environments remains limited, and reaggregation over time has been reported [18]. In contrast, the stable dispersion of PEDOT:PSS is maintained by the negatively charged sulfonate groups of the PSS chains, which promote electrostatic repulsion between particles and facilitate hydration layer formation [19]. Direct measurement of the particle properties supported this interpretation: dynamic and electrophoretic light scattering showed that the PEDOT:PSS particles were an order of magnitude smaller than the PANI particles, with a much lower polydispersity index and a strongly negative zeta potential, whereas the PANI particles were large, broadly distributed and only weakly positively charged (Figure S1). The large negative surface charge of PEDOT:PSS provides the electrostatic repulsion required for a stable aqueous dispersion, and may also influence protein adsorption at the cell-material interface. These differences in dispersibility were directly reflected in the mechanical and hydration behaviors of the resulting hydrogels. The concentration-independent G′ observed in the PANI groups is consistent with the inability of aggregated particles to reinforce the GelMA network effectively, whereas the concentration-dependent G′ increase in the PEDOT:PSS groups is likely attributable to intermolecular interactions between the negatively charged sulfonate groups of PSS and positively charged amino acid residues in GelMA, such as arginine and lysine [20]. Because PEDOT:PSS carries no polymerizable group, covalent linkage to the GelMA network during photocrosslinking is not expected; the magnitude of the increase in G′, together with the accompanying rise in loss tangent, is nevertheless consistent with the possibility that the polyanionic PSS chains act as multivalent physical crosslinkers between cationic sites on neighboring GelMA chains, which would reinforce the network while introducing additional water-binding sulfonate groups. Distinguishing such ionic junctions from the covalent network, for example, by comparing swelling in media of different ionic strengths, would be a useful extension of the present work. The swelling behavior further reflected these network structural differences: PANI-incorporated hydrogels showed an irregular concentration-dependent change in swelling ratio, consistent with locally loosened network regions due to particle aggregation, whereas PEDOT:PSS-incorporated hydrogels showed a more systematic increase attributable to expanded interchain spacing and enhanced hydrophilicity from the PSS sulfonate groups. The 0.2% formulation maintained structural integrity and appropriate mechanical properties without excessive network disruption in both materials, supporting its use in the subsequent cell studies. The cell studies were accordingly conducted at this single loading, since the aim here was to compare the two polymers within one platform rather than to resolve a concentration response; the latter is the subject of ongoing work.

The electrical characterization showed that both polymers produced a small increase in the conductance of the hydrogels, which reached significance for PANI. The size of that increase is itself informative. When a conductive polymer forms a continuous phase within a hydrogel, the conductivity rises far above that of the matrix alone: a PANI/poly(ethylene glycol) hydrogel carrying percolating PANI micropatterns reaches tens of mS/cm [17], and a pure PEDOT:PSS nanofibril hydrogel reaches the order of 10 S/cm in physiological saline [19]. Measured against such increases, the change produced here by incorporating PANI or PEDOT:PSS was a small fraction of the baseline value. The measurements are therefore consistent with dispersed polymer domains that remain electrically isolated within the matrix, with the polymer loading lying below the threshold required for percolation, and with bulk transport still carried predominantly by ionic conduction through the hydrated GelMA network; although the specimens were prepared with deionized water rather than PBS to reduce their mobile ion content, a hydrated polyelectrolyte network retains counterions and residual ionic conduction is therefore not eliminated. In culture medium, whose ionic strength is far higher than that of the deionized water used for the measurement, ionic conduction is expected to dominate even more completely, so the small electronic contribution resolved here is likely to be diluted further under the conditions the cells experienced. The measurement is therefore intended to compare the intrinsic electrical contribution of the two polymers within the matrix rather than to reproduce the electrical environment of the culture, which argues further against attributing the biological differences to it. Ionic conduction of this kind also dominates measurements on neural tissue itself, whose reported conductivity varies substantially with the measurement method and the state of the tissue [21].

Two observations argue against attributing the divergent biological outcomes to this residual electrical difference. First, no external electrical stimulation was applied in this study, so there is no evident route by which a passive, sub-percolation difference in this magnitude would act on the cells. Second, the ordering is inverted with respect to the biological outcome: the PANI groups were the more conductive at every concentration, yet PANI gave the poorer cytocompatibility and appeared to support the least neurite outgrowth.

A single structural feature is consistent with both observations. Below the percolation threshold, clustering of a conductive filler can create local particle-to-particle contacts that shortcut short segments of the matrix, whereas uniformly dispersed particles remain separated. The aggregation of PANI documented above is therefore consistent with its slightly higher conductance, while the same aggregation produces the heterogeneous, mechanically uneven interface to which the cells responded poorly. The electrical gain and the biological penalty in the PANI groups plausibly share a common origin in the dispersion state of the incorporated polymer.

This does not imply that the electrical environments experienced by cells at the material interface were equivalent. Spencer et al. reported a concentration-dependent decrease in impedance for GelMA/PEDOT:PSS hydrogels [14], indicating that uniformly dispersed conductive domains can alter interfacial charge transfer even where steady-state bulk conduction changes little. The uniform distribution of PEDOT:PSS may therefore present different local charge transfer characteristics at the cell-material interface from the heterogeneous distribution of PANI, even though the bulk conductance differences were small and ordered in the opposite direction.

In cytocompatibility evaluation, the reduced cell activity observed in the PANI 0.2% group is likely multifactorial. Previous studies have reported that PANI cytotoxicity is primarily attributable to residual impurities from synthesis and processing, including residual dopant species and low-molecular-weight byproducts, rather than to the PANI backbone itself, and that thorough purification is critical to obtaining a cytocompatible form [22]. In the present study, complete removal of the CSA dopant and DMF solvent cannot be guaranteed despite repeated washing steps. Additionally, the non-uniform dispersion of PANI particles within the GelMA matrix, as confirmed by SEM, may have introduced localized mechanical heterogeneity that adversely affected cell behavior. The precise relative contribution of each of these factors warrants further investigation. In contrast, PEDOT:PSS has been consistently reported to exhibit superior biocompatibility compared with other conductive polymers including PANI, functioning effectively under physiological conditions without requiring acidic dopants or organic solvents [23]. The comparable cell activity between the GelMA and PEDOT:PSS 0.2% groups demonstrates that the one-step fabrication approach is sufficient to maintain a cell-compatible culture environment without additional purification beyond standard washing. The lower metabolic activity observed across all hydrogel groups relative to TCP is consistent with the well-established role of substrate stiffness in YAP/TAZ-mediated mechanotransduction, whereby reduced stiffness on hydrogel substrates shifts cells away from a proliferative state [24]. Importantly, Ozgun et al. demonstrated that this reduction in proliferation on soft substrates in SH-SY5Y cells coincides with enhanced expression of neuronal differentiation markers, suggesting that the soft hydrogel microenvironment may promote a shift toward a differentiation-competent state [25].

In the neuronal differentiation experiments, the co-detection of βIII-tubulin, MAP2, and synaptophysin at day 6 in the PEDOT:PSS 0.2% group, which together span markers of early neuronal commitment, morphological maturation, and synaptic stages, suggests a more advanced overall differentiation state than in the other groups. Because these markers were assessed at a single time point, they reflect the range of differentiation stages present rather than a temporally resolved progression. βIII-tubulin expression throughout the neurites and the formation of intercellular networks indicated not only that early differentiation was initiated but also that cells extended and integrated their processes effectively, and the additional detection of MAP2 along elongated neurite-like structures was consistent with progression toward a morphologically mature neuronal phenotype [26]. The detection of synaptophysin, a presynaptic vesicle protein, at regions of cell–cell contact and along neurite projections is consistent with the presence of presynaptic components, although functional synaptic activity was not assessed in this study [27]. The weaker differentiation that appeared in the PANI 0.2% group, lower even than that in the GelMA group, likely reflects the compounding effects of residual cytotoxicity and the mechanically heterogeneous microenvironment created by particle aggregation. Differentiation was characterized here at the level of marker localization and morphology; functional readouts such as electrophysiological recording lie beyond the scope of this material comparison and are planned for subsequent work.

Taken together, these findings indicate that the more extensive differentiation that appeared in the PEDOT:PSS 0.2% group cannot be explained by the bulk electrical properties of the hydrogels, since the conductance differences between groups were small and, where they existed, favored the group that performed less well. Rather, cellular responses in GelMA-based conductive hydrogel platforms appear to be associated with a combination of material properties, including polymer dispersibility, surface chemistry, and mechanical behavior. The uniform distribution of PEDOT:PSS may provide a more homogeneous cell-material interface, supporting more consistent cell adhesion, morphological cues, and local biochemical signaling, and the negatively charged sulfonate groups of PEDOT:PSS together with its high hydrophilicity are known to influence protein adsorption and integrin-mediated signaling, which in turn regulate cytoskeletal organization and differentiation-related gene expression [19]. The higher storage modulus of the PEDOT:PSS 0.2% hydrogels may also have provided a mechanical cue favorable to neuronal differentiation, consistent with reports that substrate stiffness in the range of a few hundred Pa modulates neurite outgrowth and neuronal marker expression in SH-SY5Y and related neural cells [28,29,30]. It should be noted that reported stiffness values for central nervous system tissue span a wide range, because they depend strongly on the measurement modality, the length scale probed and the state of the tissue, so that a single representative value cannot be assigned; the hydrogels examined here (Table 1) lie at the softer end of that reported spectrum, which is the regime most consistently associated with neuronal survival and neurite outgrowth [2,25]. Importantly, because the PEDOT:PSS 0.2% group differed from the GelMA group in stiffness, surface chemistry, and dispersion simultaneously, the present design cannot isolate the individual contribution of each factor. That stiffness alone is not decisive is further supported by the comparable storage moduli of the PANI 0.2% and GelMA groups (325 and 313 Pa, respectively) despite their markedly different differentiation outcomes, which underscores the additional roles of surface chemistry, dispersion uniformity, and cytocompatibility. A practical design implication follows. Conductive hydrogels for neural tissue engineering are commonly formulated by incorporating PEDOT or PANI into a GelMA-based matrix [14,15,31]. At the loadings that a one-step, cell-compatible route can accommodate, however, the incorporated polymer does not form a percolating electronic network, so what the cells encounter is not conductivity but the interface the polymer creates. The two requirements then act on the same variable in opposite directions: electronic percolation requires particle-to-particle contact, whereas a uniform cell-material interface requires that the particles remain separated. Incorporating more of a polymer that aggregates therefore buys a small electrical gain at a disproportionate biological cost. On this view the primary selection criterion for such systems may be not the intrinsic conductivity of the conductive polymer but the stability of its dispersion and the surface chemistry it presents, which is where PEDOT:PSS outperformed PANI in this study. It also follows that a benefit attributed to conductivity should be supported by a direct measurement of it and, where an electrical mechanism is claimed, by applied stimulation.

The one-step dispersion route also proved compatible with cell encapsulation. SH-SY5Y cells mixed into the precursor and photocrosslinked in situ remained largely viable after 24 h in all groups (Figure 6), confirming that the photogelation conditions used here are cell-compatible and that the platform is not restricted to seeding cells onto preformed hydrogels. The constructs were examined at a single early time point. Because the incorporated polymers are not biodegradable, extending these constructs to longer-term three-dimensional culture and following the degradation and structural stability of the composite matrix over time define the next stage of development for this platform, ahead of any in vivo application.

The present study establishes the material basis of this comparison, and the electrical dimension of the platform defines the work that follows from it. Because the polymers were dispersed here to confer electroactivity, applying external electrical stimulation to these hydrogels and characterizing the cell-material interface electrochemically are the direct extensions of the results reported above. Together with analysis of downstream mechanotransduction and adhesion signaling, these studies will establish how the material properties identified here translate into differential neuronal responses.

## 3. Conclusions

This study demonstrated that conductive hydrogels can be fabricated in a single step by dispersing pre-polymerized PANI particles or PEDOT:PSS nanoparticles into a GelMA precursor and UV photocrosslinking, avoiding the toxic monomers and organic solvents required by conventional in situ polymerization. PEDOT:PSS was more uniformly integrated than PANI, which aggregated at higher concentrations, and these differences in dispersibility were reflected in the microstructure, mechanical, and swelling behaviors of the hydrogels. Although the incorporated polymers raised the conductance of the hydrogels only slightly, with the increase reaching statistical significance only for PANI, and did so most in the group that performed least well biologically, the biological outcomes diverged markedly: the PEDOT:PSS 0.2% group maintained cytocompatibility comparable to GelMA, and neurite outgrowth appeared most extensive in these hydrogels, with co-detection of βIII-tubulin, MAP2, and synaptophysin at day 6, whereas PANI reduced metabolic activity and appeared to limit neurite outgrowth. These results indicate that the biological performance of GelMA-based conductive hydrogels is associated not with bulk conductivity but with a combination of polymer dispersibility, surface chemistry, and mechanical properties, and suggest that at the loadings a cell-compatible one-step route permits, such hydrogels can be designed more effectively from the dispersion state of the conductive phase than from the intrinsic conductivity of the polymer incorporated.

## 4. Materials and Methods

### 4.1. Materials

All chemicals and solvents were used as received from the manufacturer unless otherwise specified. Dulbecco’s Phosphate-buffered saline (DPBS) was purchased from SolBio (Seoul, Republic of Korea). Dimethyl sulfoxide (DMSO) and N,N-Dimethyl Formamide 99.5% (DMF) were purchased from Duksan (Ansan, Gyeonggi, Republic of Korea). Fetal bovine serum (FBS) and phosphate-buffered saline (PBS, pH 7.4) were purchased from Gibco (Grand Island, NY, USA). Minimum Essential Medium (MEM), Penicillin-streptomycin (100 X) and Trypsin-EDTA solution (1X) were purchased from WelGene (Daegu, Republic of Korea). LIVE/DEAD™ Viability/Cytotoxicity Kit, Human/Mouse/Rat BDNF Recombinant Protein, paraformaldehyde, Goat anti-Rabbit IgG (H + L) Cross-Adsorbed Secondary Antibody, Phospho-MAP2 (Ser136) Polyclonal Antibody, Beta III Tubulin Recombinant Rabbit Monoclonal Antibody (HL1709), and Synaptophysin Monoclonal Antibody (SP11) were purchased from Thermo Fisher Scientific (Waltham, MA, USA). Polyaniline (emeraldine salt), (1R)-(−)-10-Camphorsulfonic acid (CSA), 2-Hydroxy-4-(2-hydroxyethoxy)-2-methylpropiophenone (Irgacure2959), Retinoic acid (RA), Triton X-100, bovine serum albumin (BSA), and 4′,6-diamidino-2-phenylindole (DAPI) were purchased from Sigma-Aldrich (Flanders, NJ, USA). Celluid GM 300 (GelMA) was purchased from CLECELL (Seoul, Republic of Korea). Cell Counting Kit-8 (CCK-8) was purchased from GLPBIO (Montclair, CA, USA). Clevios PH 1000 (PEDOT:PSS) was purchased from NANOPURITECH (Hwaseong, Gyeonggi, Republic of Korea).

### 4.2. Fabrication of Conductive Hydrogels

PANI was supplied in powder form, and a doping process was performed to improve its dispersibility and electrical conductivity [17]. Briefly, 0.01 g of PANI and 0.01 g of CSA were added to 1 mL of DMF and stirred at 60 °C for 30 min. After stirring, the solution was centrifuged at 10,000 rpm for 10 min, and the supernatant was discarded. The resulting pellet was washed twice with deionized water to remove residual DMF and free CSA, with each wash followed by centrifugation at 10,000 rpm for 10 min. The washed pellet was then resuspended in 1 mL of PBS by vortexing and pipetting to obtain a 1% *w*/*v* doped PANI solution. For PEDOT:PSS, an aqueous dispersion with a solid content of 1.0–1.3% *w*/*v* was used directly as the 1% *w*/*v* PEDOT:PSS solution. Both solutions were sonicated for 30 min prior to use to improve dispersion homogeneity.

To prepare the conductive hydrogel precursor solutions, GelMA, a 10% *w*/*v* Irgacure 2959 stock solution in DMSO, and either the PANI or PEDOT:PSS solution were added to PBS and mixed by brief vortexing and pipetting while avoiding bubble formation. All precursor solutions contained 5% (*w*/*v*) GelMA and 0.1% (*w*/*v*) Irgacure 2959, with PANI or PEDOT:PSS added at 0.1, 0.2, or 0.3% (*w*/*v*); the GelMA group contained no conductive polymer.

Disk-shaped hydrogels with a diameter of 20 mm and a thickness of 1 mm were fabricated for physicochemical characterization. A silicone mold was attached to a quartz glass plate (Anton Paar, Graz, Austria; diameter: 65 mm; thickness: 4 mm; item No. 134001), and the precursor solution was cast into the mold using an excess volume of more than 300 µL. Another quartz glass plate was carefully placed on top to prevent bubble formation. The assembly was then positioned at the center of a custom-built UV irradiation system and exposed to UV light (wavelength: 365 nm; average intensity: 3.5 mW/cm^2^; total dose: 1.05 J/cm^2^) simultaneously from the top and bottom for 5 min. For cell experiments, 100 µL of the precursor solution was cast into each well of a 48-well plate and irradiated under the same UV conditions for 3 min (total dose: 0.63 J/cm^2^) to form a thin hydrogel coating on the well surface.

### 4.3. Physicochemical and Electrical Characterization

The particle size, polydispersity index and zeta potential of the PANI and PEDOT:PSS dispersions were measured by dynamic and electrophoretic light scattering; the procedure and the resulting data are given in the Supplementary Materials.

The microstructural morphology of the freeze-dried hydrogels was examined by scanning electron microscopy (SEM) (SU8600, Hitachi, Tokyo, Japan; Smart Materials Research Center for IoT supported by the Korea Basic Science Institute, NFEC-2023-02-285654). Prior to imaging, hydrogel samples were rapidly frozen in liquid nitrogen, transferred to a deep freezer at −80 °C for 4 h, and subsequently lyophilized for 24 h. Cross-sectional surfaces were obtained by sectioning the freeze-dried samples with a microtome blade. The samples were sputter-coated with Pd-Pt prior to imaging at magnifications of ×400 and ×1000.

Pore size was quantified from the ×400 SEM images using ImageJ (version 1.54g; National Institutes of Health, Bethesda, MD, USA). The scale was calibrated separately for each micrograph from its own scale bar. Pores were sampled with an unbiased counting frame, and the longest axis of every pore satisfying the inclusion criteria was measured without selective sampling.

The viscoelastic properties of the hydrogels were evaluated using a rheometer (MCR92, Anton Paar, Graz, Austria) equipped with a 25 mm parallel plate geometry. Disk-shaped hydrogels (diameter: 20 mm; thickness: 1 mm) were loaded onto the measurement platform, and a frequency sweep was performed from 0.1 to 10 Hz at a fixed strain of 1% to obtain the storage modulus (G′) and loss modulus (G″). Three independent specimens were measured for each group, and all measurements were performed at 37 °C.

The swelling behavior of the hydrogels was assessed by gravimetric analysis. Fabricated hydrogels were washed three times with PBS (30 min each) to remove residual unreacted components, then immersed in DPBS and maintained at 37 °C. At designated time points (0, 1, 4, 8, 12, and 24 h), the hydrogels were removed, surface moisture was gently blotted, and the swollen weight (w_t_) was recorded. The hydrogels were subsequently lyophilized for 24 h, and the dry weight (w_0_) was measured. The swelling ratio was calculated according to Equation (1):

(1) Swelling ratio (%) = (w_t_ − w_0_)/w_0_ × 100%

The electrical properties of the hydrogels were evaluated by direct-current resistance measurement. Hydrogel precursor solutions were prepared as described in Section 4.2, except that deionized water was used as the solvent in place of PBS in order to reduce the mobile ion content of the specimens. The solutions were cast onto glass substrates patterned with electrodes and photocrosslinked in place. Two probe tips of a probe station were brought into contact with the electrode pads at either end of the specimen, a source current of 0.1 mA was supplied, and the resistance was recorded; this current lies within the range commonly applied for such measurements. Conductance was calculated as the reciprocal of the measured resistance. Because the specimens were formed as coatings on the electrodes, the current path length and the cross-sectional area could not be determined with sufficient accuracy to convert the measurements into conductivity, and the results are therefore reported as resistance and conductance. Three independent specimens were measured for each group.

### 4.4. Cell Culture and Differentiation

The hydrogel-coated wells were sterilized by immersion in 70% ethanol for 3 h, followed by washing with DPBS for 1 h and overnight incubation in culture medium prior to cell seeding. SH-SY5Y human neuroblastoma cells (Korean Cell Line Bank, Seoul, Republic of Korea) were maintained in Minimum Essential Medium (MEM) supplemented with 10% heat-inactivated fetal bovine serum (HI-FBS) and 1% Penicillin-Streptomycin. Cells were cultured at 37 °C in a 5% CO_2_ atmosphere and passaged upon reaching approximately 80% confluence.

For cytocompatibility assessment, cells were seeded onto hydrogel-coated 48-well plates at a density of 2 × 10^4^ cells/well in growth medium and cultured for up to 7 days. Uncoated wells of the same tissue culture plate were seeded in parallel and served as the TCP control.

For differentiation experiments, cells were seeded at a density of 4 × 10^4^ cells/well and cultured in growth medium for 48 h. The medium was then replaced with differentiation medium 1, consisting of MEM supplemented with 1% HI-FBS, 1% Penicillin-Streptomycin, and 10 µM retinoic acid (RA). Plates were wrapped in aluminum foil to minimize light exposure and prevent RA photodegradation. After 72 h, the medium was replaced with differentiation medium 2, consisting of MEM supplemented with 1% HI-FBS, 1% Penicillin-Streptomycin, and 50 ng/mL BDNF, and cells were cultured for an additional 72 h.

### 4.5. Cell Encapsulation in Conductive Hydrogels

To encapsulate SH-SY5Y cells within the conductive hydrogels, the precursor solution was prepared as described in Section 4.2, except that culture medium was used as the solvent in place of PBS. A cell suspension was gently mixed with the precursor solution, and 10 µL of the cell-containing precursor (7 × 10^4^ cells) was cast into each well of a 48-well plate and exposed to UV light under the conditions described in Section 4.2 to form cell-laden hydrogels. The constructs were cultured in growth medium for 24 h, after which cell viability was assessed by Live/Dead staining as described in Section 4.6.

### 4.6. Cytocompatibility Assessment

Cell viability was assessed using the LIVE/DEAD Viability/Cytotoxicity Kit. On days 1, 4, and 7 of culture, the medium was aspirated and cells were incubated with 500 µL of staining solution containing 2 µM calcein AM and 4 µM ethidium homodimer-1 for 1 h at 37 °C. Following incubation, cells were washed gently with DPBS and imaged using a fluorescence microscope (EVOS M5000, Invitrogen, Waltham, MA, USA).

Metabolic activity was quantitatively evaluated using the CCK-8 assay on days 1, 4, and 7, with three independent biological replicates per group at each time point. At each time point, 100 µL of CCK-8 solution was added to each well containing 1 mL of fresh culture medium, and plates were incubated for 4 h at 37 °C. Subsequently, 200 µL of the resulting solution was transferred to a 96-well plate, and absorbance was measured at 450 nm using a microplate reader (Epoch, BioTek, Winooski, VT, USA). Acellular hydrogel-coated wells of each composition (GelMA, PANI 0.2% and PEDOT:PSS 0.2%), and uncoated wells for the TCP control, were incubated with CCK-8 reagent in parallel under identical conditions at every time point, and their absorbance was subtracted from that of the corresponding cell-containing wells as material-specific blanks before normalization. Absorbance values were then expressed as a percentage of the mean of the GelMA group at the same time point, where Asample is the absorbance of an individual well and AGelMA is the mean absorbance of the GelMA group at that time point. The relative metabolic activity was calculated according to Equation (2):

(2) Relative metabolic activity (%) = A_sample_/A_GelMA_ × 100%

### 4.7. Immunofluorescence Staining

On day 6 of differentiation, cells were fixed with 4% paraformaldehyde for 20 min, permeabilized with 0.1% Triton X-100 for 10 min, and blocked with 3% BSA in DPBS for 2 h with mild shaking. Cells were then incubated overnight at 4 °C with the following primary antibodies diluted 1:250 in 3% BSA: rabbit anti-phospho-MAP2 (Ser136) polyclonal antibody, rabbit anti-βIII-tubulin monoclonal antibody, and rabbit anti-synaptophysin monoclonal antibody. After washing three times with PBS (5 min each), cells were incubated with Alexa Fluor 488 goat anti-rabbit IgG secondary antibody (1:500 in 3% BSA) for 2 h at room temperature in the dark. Cells were washed three times again with PBS, and nuclei were counterstained with DAPI (1 µg/mL) for 10 min. Fluorescence images were acquired using a fluorescence microscope (EVOS M5000, Invitrogen, Waltham, MA, USA).

### 4.8. Neurite Quantification

The descriptive neurite counts and lengths shown in Figure S2 were obtained from a representative βIII-tubulin image of each group as described in the Supplementary Materials.

### 4.9. Statistical Analysis

All data are presented as mean ± standard deviation. The number of replicates for each measurement is given in the corresponding Methods subsection and figure legend. Statistical analysis was performed using one-way analysis of variance (ANOVA) followed by Tukey’s honestly significant difference (HSD) post-hoc test, using GraphPad Prism 11 (GraphPad Software, San Diego, CA, USA). For the CCK-8 assay, absorbance values were normalized to the mean of the GelMA group at the same time point and comparisons were made within each time point. Pore size was obtained from a single representative image per group and is presented as a distribution without statistical testing; the descriptive neurite measurements in Figure S2 were likewise not subjected to statistical comparison. Differences were considered statistically significant at *p*  <  0.05 (*), *p*  <  0.01 (**), and *p*  <  0.001 (***).

## Figures and Tables

**Table 1:** Viscoelastic properties of the conductive GelMA hydrogels obtained from frequency sweep rheometry at 37 °C. Values are mean ± standard deviation.

Group	G′ (Pa)	G″ (Pa)	Tan δ
GelMA	312.58 ± 6.46	5.42 ± 2.68	0.017 ± 0.009
PANI 0.1%	354.96 ± 3.02	5.05 ± 2.80	0.014 ± 0.008
PANI 0.2%	325.03 ± 9.09	5.84 ± 2.06	0.018 ± 0.006
PANI 0.3%	317.74 ± 3.58	6.85 ± 2.88	0.022 ± 0.009
PEDOT:PSS 0.1%	347.86 ± 21.16	10.93 ± 1.99	0.031 ± 0.006
PEDOT:PSS 0.2%	586.62 ± 12.86	22.56 ± 3.33	0.038 ± 0.006
PEDOT:PSS 0.3%	925.47 ± 30.31	43.02 ± 4.41	0.047 ± 0.005

**Table 2:** Electrical resistance and the corresponding conductance of the conductive GelMA hydrogels. Values are the mean ± standard deviation of three independent specimens.

Group	Resistance (kΩ)	Conductance (µS)
GelMA	45.10 ± 0.34	22.17 ± 0.17
PANI 0.1%	40.98 ± 1.62	24.44 ± 0.96
PANI 0.2%	39.33 ± 0.41	25.43 ± 0.27
PANI 0.3%	38.50 ± 1.43	26.01 ± 0.98
PEDOT:PSS 0.1%	43.97 ± 0.35	22.77 ± 0.19
PEDOT:PSS 0.2%	43.58 ± 1.65	22.97 ± 0.85
PEDOT:PSS 0.3%	43.00 ± 3.10	23.34 ± 1.65

## Data Availability

The data generated or analyzed during this study are available from the corresponding authors on reasonable request.

## Supplementary Materials

The following supporting information can be downloaded at: https://www.mdpi.com/article/10.3390/gels12100892/s1, Figure S1: Particle size, polydispersity index and zeta potential of the PANI and PEDOT:PSS dispersions; Figure S2: Descriptive neurite counts and lengths measured from a representative βIII-tubulin image of each group.
