# Supplementary material for: Comparison of PANI- and PEDOT:PSS-Incorporated GelMA Hydrogels for Neuronal Differentiation of SH-SY5Y Cells
Source: Gels. 2026 Oct 2;12(10):892. doi: 10.3390/gels12100892 (PMC13704704; doi:10.3390/gels12100892)
Supplement: Supplementary file 1 [file gels-12-00892-s001.zip › gels-4578377-supplementary.pdf]

Supplementary Materials

Comparison of PANI- and PEDOT:PSS-Incorporated GelMA Hydrogels for Neuronal Differentiation of SH-SY5Y Cells

Gayeon Pyo, Ha Som Yu, Yeontae Kang, Hansol Lee * and Hyun Jong Lee *

Department of Chemical and Biological Engineering, Gachon University, 1342 Seongnam-daero, Seongnam-si, Gyeonggi-do 13120, Republic of Korea

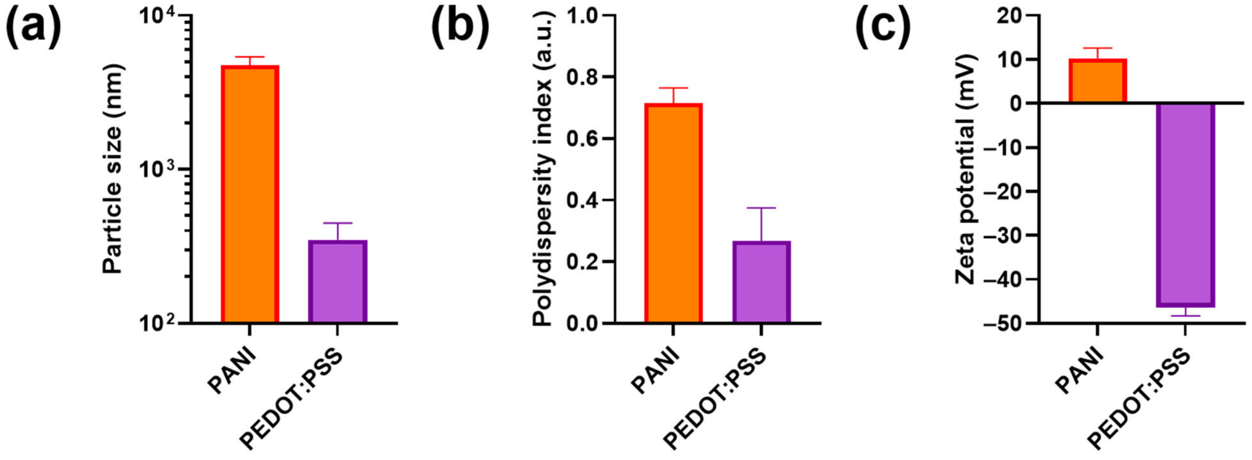

Figure S1. Particle characteristics of the PANI and PEDOT:PSS dispersions. (a) Particle size determined by dynamic light scattering (note the logarithmic axis). (b) Polydispersity index. (c) Zeta potential determined by electrophoretic light scattering. Bars are mean ± standard deviation and circles are the individual measurements (n = 3).

Methods S1. Particle characterization

The particle size and zeta potential of PANI and PEDOT:PSS were measured using an ELSZ-2000 (Otsuka Electronics, Osaka, Japan). For sample preparation, PANI and PEDOT:PSS were dispersed in triple-distilled water and diluted to final concentrations of 0.001% (w/v) and 0.005% (w/v), respectively. The resulting dispersions were analyzed by dynamic light scattering (particle size and polydispersity index) and by electrophoretic light scattering (zeta potential). Three independent measurements were performed for each material.

The PANI particles were approximately an order of magnitude larger than the PEDOT:PSS particles and had a markedly higher polydispersity index, indicating a broad and less uniform size distribution. The zeta potential of PEDOT:PSS was strongly negative, whereas that of PANI was only weakly positive, so that the electrostatic repulsion available to stabilize the dispersion is far greater for PEDOT:PSS. These measurements are consistent with the difference in dispersion state observed within the GelMA matrix.

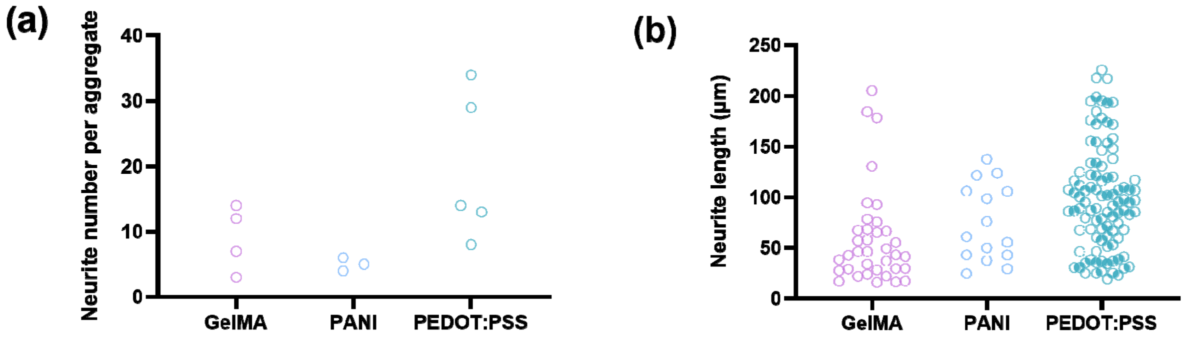

Figure S2. Descriptive neurite measurements from a representative βIII-tubulin immunofluorescence image of each group on day 6 of differentiation. (a) Number of neurites extending from each cell aggregate and (b) length of individual neurites; each circle is one aggregate in (a) and one neurite in (b). The values were obtained from a single field per group (4, 3 and 5 aggregates and 36, 15 and 99 neurites for GelMA, PANI 0.2% and PEDOT:PSS 0.2%, respectively) and therefore describe the representative images shown in Figure 5c only; they were not used for statistical comparison between groups.

Methods S2. Descriptive neurite measurement

Neurite number and length were measured from the ×4 βIII-tubulin immunofluorescence image of each hydrogel group shown in Figure 5c using ImageJ (National Institutes of Health, USA). Every cell aggregate within the field was included: the number of neurites extending from each aggregate was counted, and the length of each neurite was traced from the aggregate boundary to its tip. The TCP control was not analyzed because the densely interconnected network formed at high cell density did not allow the origin of individual neurites to be assigned. Because each group is represented by a single field, the measurements are reported only as a description of the representative images and no statistical test was applied.
